# Neuronal CD59 isoforms IRIS-1 and IRIS-2 as regulators of neurotransmitter release with implications for Alzheimer’s disease

**DOI:** 10.1186/s13195-024-01660-z

**Published:** 2025-01-07

**Authors:** Ewelina Golec, Robin Olsson, Emre Can Tuysuz, Maja Karlsson, Yasmin Serjieh, Ben C. King, Malin Wennström, Anna M. Blom

**Affiliations:** 1 Section of Medical Protein Chemistry, Department of Translational Medicine, Lund University, Malmö, 214-28 Sweden; 2 Cognitive Disorder Research Unit, Department of Clinical Sciences Malmö, Lund University, Malmö, 214-28 Sweden

**Keywords:** Alzheimer’s disease, CD59, Intracellular complement, IRIS-1, IRIS-2, SNARE, Tau hyperphosphorylation, Type 2 diabetes, Neurotransmitters release

## Abstract

We have previously demonstrated that the intracellular, non-GPI anchored CD59 isoforms IRIS-1 and IRIS-2 (Isoforms Rescuing Insulin Secretion 1 and 2) are necessary for insulin secretion from pancreatic β-cells. While investigating their expression across human tissues, we identified IRIS-1 and IRIS-2 mRNA in the human brain, though their protein expression and function remained unclear. This study shows the presence of both IRIS-1 and 2 proteins in the human brain, specifically in neurons and astrocytes. In the neuroblastoma cell line (SH-SY5Y), both isoforms are intracellular, and their expression increases upon differentiation into mature neurons. Silencing IRIS-1 and 2 in SH-SY5Y cells reduces the SNARE complex formation, essential for synaptic vesicle exocytosis, leading to a reduction in noradrenaline secretion. Notably, we observed diminished expression of neuronal IRIS-1 and 2 in patients with Alzheimer’s disease (AD) and non-demented individuals with type 2 diabetes (T2D). In SH-SY5Y cells, knockdown of all isoforms of CD59 including IRIS-1 and 2 not only elevates phosphorylated tau but also increases cyclin-dependent kinase 5 (CDK5) expression, known promoter of hyperphosphorylation and accumulation of tau, a key pathological feature of AD. Additionally, we found that prolonged exposure to high glucose or cytokines markedly reduces the expression of IRIS-1 and 2 in SH-SY5Y cells, suggesting a link between AD pathology and metabolic stress through modulation of these isoforms.

**Supplementary Information:**

The online version contains supplementary material available at 10.1186/s13195-024-01660-z.

## Significance statement

The study reveals the existence of intracellular, non-GPI anchored CD59 isoforms IRIS-1 and IRIS-2 in neurons and astrocytes within the human brain. It also demonstrates decreased expression of IRIS-1 and 2 in neurons of individuals with AD and T2D, pointing to a link between aberrant expression of IRIS isoforms and deficiencies in neurotransmitter release in patients with AD. Identifying specific IRIS-1 and IRIS-2 binding partners within the neurons and the exact mechanism leading to its reduced expression in AD could provide new therapeutic targets to delay the symptomatic progression of Alzheimer’s disease.

**Supplementary Information:**

The online version contains supplementary material available at 10.1186/s13195-024-01660-z.

## Introduction

Alzheimer’s disease (AD) is a neurodegenerative disorder accounting for approximately 70% of all dementia cases [1]. The disorder is neuropathologically characterized by the presence of senile plaques and neurofibrillary tangles (NFTs), where the former arise due to an accumulation of amyloid beta (Aβ) and the latter is a result of intra-neuronal accumulation of hyperphosphorylated tau (p-tau). These two key hallmarks are associated with substantial neuronal and synaptic loss and neuroinflammatory actions, where the pivotal role of microglia and astrocyte activation is well-established [2]. Furthermore, an increasing number of studies suggest that also the complement system is implicated in AD [3]. The terminal pathway of complement activation results in the formation of the membrane-attack complex (MAC), which via pore formation can lyse the cells. Host cells are protected from MAC-mediated damage by CD59 protein [4], a ubiquitously expressed membrane-bound complement inhibitor. Reduced levels of CD59 have been found in the hippocampus and frontal cortex of AD patients compared to non-demented controls, and Aß seems to have a direct negative impact on CD59 expression [5]. Hence, a loss of CD59 may have detrimental effects. Indeed, primary deficiency of CD59 results in the destruction of nerve tissue due to uncontrolled complement attack [6, 7] presenting as an acute, predominantly motor, demyelinating neuropathy, indistinguishable from recurrent Guillain–Barré syndrome (GBS) [6]. CD59 appears to also have other functions besides being a cell-surface attached complement inhibitor. We have recently shown that CD59 is present intracellularly in pancreatic β-cells [8, 9]. In addition, we discovered the presence of two splice variants of CD59, called IRIS-1 and IRIS-2 [10]. IRIS-1 (Ref. no.: ENST00000534312.5) lacks the canonical exon 4 encoding the GPI-anchor signal sequence. An additional 3’ exon encoding the C-terminal domain of IRIS-1 is instead derived from the adjacent uncharacterized predicted open reading frame C11orf91, which has an evolutionarily conserved position adjacent to the CD59 gene. IRIS-2 (Ref. no.: ENST00000643183.1), in contrast, has an additional exon inserted between canonical exons 3 and 4, causing a frameshift at the C-terminus; therefore, IRIS-2 has about 47% similarity to canonical CD59 and is also not GPI anchored. These transcripts are identical to the canonical CD59 at the 5’ region, and all therefore encode proteins with N-terminal signal peptides, causing entry into the endoplasmic reticulum (ER) and secretory pathway. However, we found no IRIS-1 or IRIS-2 secreted into the extracellular environment, but rather found that these proteins are retained intracellularly within the cytosol. Expression of CD59 lacking the GPI anchor sequence had the same result [9], and this was found to be dependent on the N-glycosylation site that is shared between all three isoforms. The N-linked glycan was of smaller size within the cytosolic fraction than that of cell-surface canonical CD59, indicating that it was trimmed, which typically takes place when a protein is resident within the ER without being trafficked further; the recognition of this trimmed glycan by ER-resident lectins results in retro-translocation across the ER membrane into the cytosol [11]. While this process typically leads to proteasomal degradation, the IRIS isoforms escape this and are functional within the cytosol.

Our study showed that the intracellular IRIS-1 and 2 play a crucial role in the regulation of β-cell insulin secretion [10]. This process is dependent on SNARE proteins, a large family of membrane-associated proteins, which assemble into coiled-coil bundles, pulling opposing lipid bilayers together to induce a fusion event between insulin-containing vesicles and the plasma membrane [12]. The SNARE core complex consists of vesicle-associated membrane protein 2 (VAMP2, or Synaptobrevin), plasma membrane-bound Syntaxin 1, and synaptosomal-associated protein 25 (SNAP-25). We showed that the two splice variants of CD59 interact with both VAMP2 and SNAP-25 as well as with insulin granules. In addition, in experimental studies, we demonstrated decreased expression of IRIS-1 and 2 in response to hyperglycemia, and a rescuing effect of the two CD59 splice variants on insulin secretion in CD59, IRIS-1 and 2 knockout cells, suggesting a critical role of IRIS-1 and 2 variants in SNARE-dependent release of vesicle content. Interestingly, mRNA coding for IRIS-1 and 2 are not restricted to the pancreas but are widely expressed throughout the human body, including the brain [10]. No studies have been performed to date addressing the intracellular expression and function of IRIS-1 and 2 in the brain, but considering the fact that neuronal signaling i.e., neurotransmitter release in synapses is strongly dependent on tightly regulated secretion pathway for fast stimulus-dependent cargo release [13] it is likely that the two IRIS isoforms also play a role in neurotransmission. The SNARE core complex in neurons consists of the same isoforms as in β-cells, namely VAMP2, Syntaxin 1, and SNAP-25, and these are essential for vesicle exocytosis and, thereby, for synaptic transmission [12]. Neurotransmitter release is Ca^2+^ dependent. The arrival of firing action potential leads to the depolarization of presynaptic terminals, causing transient voltage-gated Ca^2+^ channel opening, leading to a rise of Ca^2+^ influx into neurons, which binds to synaptotagmin (Syt), and induces the assembly of the SNARE complex, mediating the docking and priming of presynaptic vesicles [14]. Given that neurotransmitter release, just like insulin secretion by β-cells, is Ca^2+^ and SNARE complex dependent, it is tempting to speculate that IRIS-1 and 2 may be implicated in neurotransmission.

In this study, we show that IRIS-1 and 2 are localized mainly in neurons and astrocytes with minimal or no presence observed in microglia within the human brain. Further, silencing of all isoforms of CD59 including IRIS-1 and 2 reduced SNARE complex formation in cultured neurons (SH-SY5Y) leading to a reduction in noradrenaline secretion. Additionally, the expression of neuronal IRIS-1 and 2 was reduced in AD and non-demented T2D cases, and by glucotoxicity and prolonged cytokine exposure. Finally, IRIS-1 and 2 knockdowns increased both the expression of cyclin-dependent kinase 5, a kinase implicated in tau phosphorylation, as well as p-tau levels, potentially accelerating AD progression.

## Materials and methods

### Individuals included in the study

The presence of IRIS-1 and 2 was analyzed in Cornu Ammonis 1 (CA1) of postmortem collected hippocampal samples from cases (Netherlands Brain Bank; NBB) separated into three groups. Group 1 A (*n* = 6) included (*n* = 3) non-demented controls (NC) and (*n* = 3) clinically diagnosed and neuropathologically verified AD cases, Group 1B included (*n* = 7) clinically diagnosed and neuropathologically verified AD cases, and Group 2 (*n* = 6) consisted of (*n* = 3) non‐demented controls without T2D (NC) and (*n* = 3) clinically diagnosed non-demented T2D cases (NC-T2D). The demographics of the individuals are shown in Table 1. The presence of NFT and LB was scored according to Braak stages I-VI [11] and the Aβ plaques were scored into O, A, B, C, where O = zero, A = some, B = moderate and C = many [11]. Cases without cognitive impairments and NFT scores below III and Aβ below A were considered as NC.

### Ethical approval and patient consents

Informed consent for the use of brain tissue, plasma, and clinical data for research purposes were obtained from all subjects or their legal representatives in accordance with the International Declaration of Helsinki [15]. The tissue collection protocols were approved by the medical ethics committee of VU medical Centre in Amsterdam, the Netherlands and the Swedish Ethical Review Authority approved the study (Dnr 2016/155, 2017/717).

### Brain sample preparation

Directly after autopsy, brain samples containing hippocampus and entorhinal cortex in Group 1 A and B were immersion-fixed in paraformaldehyde (PFA) (4%) for 14–20 h. Brain samples in Group 2 were freshly frozen without cryoprotectant directly after autopsy and remained frozen until immersion-fixed in PFA (4%) for 4 h. After fixation, the samples from all groups were incubated in phosphate-buffered saline (PBS) with 30% sucrose. They were then sectioned in 40 μm free-floating sections and stored in cryoprotectants at − 20 °C until they were used for immunostaining.

### Brain samples immunostaining

For analysis of cellular localization of IRIS-1 and 2 sections from NC and AD (Group 1 A) were stained with antibodies against IRIS-1 and 2 (in-house generated rabbit antibodies against the unique C-terminal peptides existing in the novel isoforms but not in canonical CD59 [10]), together with antibodies directed against Iba-1 (microglia marker), GFAP (astrocyte marker) and NeuN (neuronal marker). The sections were incubated for 1 h with blocking solution containing 5% goat serum (Jackson Immunoresearch) and 0.25% Triton X-100 in PBS, and then incubated with rabbit-anti-IRIS-1 or rabbit-anti-IRIS-2 (Capra, #613.610 and #612.609 respectively) together with either chicken-anti-GFAP (1:500, Merck) or Mouse anti-NeuN (1:200, Proteintech) in blocking solution overnight (ON) at 4 °C. The next day the sections were washed and incubated with secondary antibodies Alexa 488 goat-anti-mouse (#A11029, Thermo Fisher Scientific) and dylight 549 goat-anti-rabbit (#DI-1549, Vector Laboratories) in blocking solution for 2 h at room temperature (RT). The Iba-1 co-staining with IRIS-1 and 2 was performed sequentially, starting with the staining against IRIS-1 or 2 according to the above instructions. The day after the sections were incubated with rabbit anti-Iba-1 (1:500, Wako) ON at 4 °C and with Alexa 488 goat-anti-rabbit (#A11029, Thermo Fisher Scientific) for 2 h the following day. To analyze the relationship between p-tau load and IRIS-1 and 2, sections (Group 1B) were stained against AT8 (phosphorylation of tau at sites Ser202, Thr205) and IRIS-1 or 2. The same protocol as above was used with mouse-anti-AT8 (1:500, Invitrogen) and the IRIS-1 and 2 antibodies as the primary antibody. Sections stained according to all the above immunofluorescence protocols, but with PBS instead of the primary antibodies were used as negative control. All stained sections were incubated in Sudan Black (1% in 70% ethanol) (Sigma-Aldrich) for 5 min before they were rinsed and mounted with a Vectashield Set mounting medium containing DAPI (Vector Laboratories).

For analysis of IRIS-1 and IRIS-2 expression in hippocampus and entorhinal cortex and quantification of the mean signal intensity of IRIS-1 and IRIS-2 immunoreactivity, sections from NC and AD (Group 1 A), NC, and T2D-NC (Group 2) were immunohistochemically stained with antibodies against IRIS-1 and 2. The sections were incubated in quenching solution (3% H_2_O_2_, 10% methanol) for 30 min, followed by Impress reagent kit blocking solution (Vector Laboratories #MP-7402) for 1 h at room temperature and then incubated with rabbit-anti-IRIS-1 or rabbit-anti-IRIS-2 in blocking solution ON at 4 °C. The following day the sections were incubated in Ig Impress reagent kit secondary anti-rabbit antibody (Vector Laboratories #MP-7401) at RT for 2 h, and then developed for 2 min in 0.25 mg/ mL diaminobenzidine and 0.012% H_2_O_2_. The sections were mounted with DPX (Sigma Aldrich, #06522).

### Analysis of IRIS-1 and 2 expression in NC, AD and T2D brains

To analyze the mean signal intensity of IRIS-1 and 2 immunoreactivity in Group 1 A, 1B and Group 2 at least 6 images were taken per case with 10–20 neurons per image. Fiji program was used for the quantifications, where a manual threshold was adjusted for each case, and region of interest (ROI) (drawn around single neurons) was selected. Around 10–12 neurons per photo were quantified where the mean value for the mean signal intensity was taken, and compared between three controls and three AD, or non-demented T2D cases. All images and quantifications were captured/ performed blindly.

### Analysis of p-tau and IRIS-1 and 2 expression (confocal microscopy)

Sections stained against AT8, IRIS-1 and IRIS-2 were analyzed using confocal microscopy. Z-stack images were taken and the maximum intensity projection was used to compile the stacks into single images using the Zen software. The images were exported as TIFF and the channels for IRIS-1/ 2 and p-tau were then quantified individually for all CA1 neurons with the Fiji software using a ROI (neurons). The ratio of IRIS-1/ 2 to p-tau was taken and categories were made according to the ratio from low, intermediate to high where < 2 was low, 2–4 intermediate and > 4 high for IRIS-1 with 0.5 lower threshold for all categories for IRIS-2. A minimum of 10 neurons per ratio category were quantified per case (*n* = 7). Due to having clustered data where neurons between cases cannot be considered independent, a linear mixed model was used to avoid pseudoreplication. To analyze the data statistically, R 4.3.3. was used with the Ime4 package. The following model specifications were used: Imer (IRIS-1/ 2 ∼ ptau + (1| case), REML = FALSE, control = lmerControl (‘bobyqa’), data = MixedModel). A likelihood ratio test (LRT) was used to see if the full model was significantly better than the null model (Alfa = 0.05), and the 95% confidence interval was extracted to get a sense of the variation. The model assumes that the residuals are normally distributed, which was assessed by plotting them against a normal distribution curve. The LRT assumes a Chi-square distribution, and that the restricted (residual) maximum likelihood (REML) is set to false. We assumed fixed slopes and random intercepts for the linear mixed model since that is the standard, and we had no theoretical reason to believe otherwise. The control used was BOBYQA, which performs a derivative-free bound-constrained optimization using an iteratively constructed quadratic approximation for the objective function, which is used to optimize the models performance.

### Localization of IRIS-1 and 2 in astrocytes, microglia and neurons

Localisation of IRIS-1 and IRIS-2 in microglia (Iba-1 positive cells)) and astrocytes (GFAP-positive cells) and neurons (NeuN) were analyzed by capturing Z-stack images, which were compiled into a single image using the maximum intensity projection in Zen software, and thereafter adjusted for clarity. For analysis of IRIS-1 and 2 in astrocytes and microglia at least 10 images were taken from three cases in Group 1 A (in total *n* = 30), while the presence of IRIS-1 and 2 in neurons were analyzed in one NC case.

**Table 1:** Clinical diagnosis, sex, age, neuropathological assessment, cause of death and postmortem delay of cases included in the study

Groups		Clinical diagnosis	Sex	Age (years)	Neuropat. v (NFT/Aβ/LB)	Cause of death	PMD
Group 1 A		NC.1	M	72	2/O/0	Pneumonia	11:00
		NC.2	F	55	1/O/0	Euthanasia (Chronic pain)	7:30
		NC.3	F	75	1/A/0	Euthanasia (Heart failure)	6:57
	Group1B	AD.1	M	94	5/C/0	End-stage dementia	6:20
		AD.2	F	81	4/C/0	Pyelonephritis	6:30
		AD.3	M	68	5/C/0	Euthanasia (AD)	6:32
		AD.4	M	64	6/C/0	Euthanasia (AD)	6:65
		AD.5	F	91	5/C/5	CVA	6:56
		AD.6	M	72	6/C/0	Pneumonia	6:12
		AD.7	M	82	5/C/0	AD	5:95
Group 2		NC.1	M	75	1/O/0	Cardiac arrest	7:10
		NC.2	M	70	1/O/3	Pneumonia	6:59
		NC.3	F	60	0/O/0	Mammary carcinoma	6:58
		NC-T2D.1	F	60	0/O/0	Decreased intake	6:30
		NC-T2D.2	F	68	1/O/0	Euthanasia (T2D)	4:30
		NC-T2D.3	M	78	1/O/0	Euthanasia (T2D)	6:41

NC = non-demented controls, AD = Alzheimer’s disease, NC-T2D = non-demented type 2 diabetes, NFT = Neurofibrillary tangles, LB = Lewy bodies, Aβ = amyloid beta. F = female, M = male. PMD = post mortem delay

### SH-SY5Y cell line

SH-SY5Y cells is a neuroblastoma cell line derived from a 4-year-old female patient with metastatic bone tumor [16]. Cells were regularly tested for Mycoplasma (Eurofins Genomic, detection based on Sanger sequencing), and were cultured in a humidified chamber with 5% CO_2_ at 37 °C. SH-SY5Y cells can be differentiated into mature neuron-like phenotype characterized by presence of neuronal markers using BDNF and retinoic acid. In brief, SH-SY5Y cells (Sigma-Aldrich, St. Louis, MO, USA) were cultured in Eagle’s minimum essential medium (EMEM, Sigma, #M2279), and Ham’s F-12 nutrient mix (Sigma, #N4888), supplemented with nonessential amino acids (Sigma, #M7145), 15% fetal bovine serum (ATCC), 500U L-Glutamine (Cytiva), penicillin (100 U/ ml) and streptomycin (100 µg/ ml). Cell culture plates were coated with 10 g/ ml of poly-D-lysine, 1 h prior to cells seeding.

### SH-SY5Y cells differentiation into mature neurons

Culture medium was replaced 1–2 days after seeding with phenol free Dulbecco’s Modified Eagle Medium (DMEM), supplemented with 5% fetal bovine serum (ATCC) and 10 µM all-trans-retinoic acid (ATRA). Cells were grown in an ATRA-containing medium for 3 days and the medium was refreshed every day. The medium was replaced with a second differentiation medium, containing phenol-free neurobasal medium (containing GlutaMAX, 200 mM), 1x N2-supplement and 50 ng/ml brain-derived neurotrophic factor (BDNF). Cells were grown in BDNF-containing neurobasal medium for a minimum of 4 days, and the medium was refreshed daily. Differentiation was monitored microscopically via morphological assessment of neurite outgrowth, as well by real-time PCR using the neural markers.

### RT-PCR

RNA was isolated from differentiated and non-differentiated SH-SY5Y cells using RNA purification kit (Qiagen, #74136). cDNA was synthesized using oligo-dT primers and Superscript IV (Invitrogen) according to standard protocol, and amplicon amplified using DreamTaq Green PCR Master mix (Thermo Scientific) using annealing temperature 62 °C for 30 s for CD59, IRIS-1 and IRIS-2, and 54 °C for 30 s for glyceraldehyde-3-phosphate dehydrogenase (GAPDH). For all the amplicons extension time of 30 s was used, with final extension of 15 min. Samples were separated on 1.2% agarose gel and visualized using SybrSafe reagent. GAPDH was used as a control. For detection of total human CD59 transcript primers 1 and 2 were used. To detect human IRIS-1, primers 1 and 3 were used, and to detect human IRIS-2, primers 4 and 2 were used.

Primer 1: Forward; ATC ACA ATG GGA ATC CAA GGA GGG.

Primer 2: Reverse; CTC TCC TGG TGT TGA CTT AGG G.

Primer 3: IRIS-1 Reverse; CTC AGG AGA GAG AGG CCG AC.

Primer 4: IRIS-2 Forward; AGT TGG GAT ATC ACT ATG TTG CCC.

### Semi-quantitative RT-PCR

The cDNA was amplified with TaKaRa Ex Premier^™^ DNA polymerase 2x PCR Master mix (TaKaRa) with the same programs for IRIS-2/CD59 and GAPDH but with an extension temperature of 68 °C, a denaturation temperature of 98 °C for 10 s, and a final extension of 5 min. For IRIS-1, the annealing temperature was 57 °C for 10 s. Samples were separated on 1.1% agarose gels and visualized using SybrSafe reagent.

### Western blots

Cells were lysed in RIPA lysis buffer (150 mM NaCl, 50 mM Tris-HCl, pH 7.5, 1% NP-40, and 0.5% deoxycholate) with addition of protease and phosphatase inhibitors (Thermo Scientific). Proteins were resolved on 15% SDS-PAGE gel (home-made), or 4–20% Precast Protein gels (Bio-Rad) under reducing conditions unless specified otherwise, transferred onto PVDF membrane using Trans-Blot Turbo system (Bio-Rad) and blocked with Quench buffer (3% fish gelatin in Immunowash: 50 mM Tris-HCl, 150 mM NaCl, 0.1% Tween 20, pH 8.0), 5% BSA in immunowash (Sigma-Aldritch #126579), or EveryBlot (Bio-Rad). The membranes were probed with suitable antibodies followed by the addition of appropriate HRP-conjugated secondary antibody (Dako) specifically stated in each subsection, and development with ECL reagent (Santa Cruz Biotechnology).The membranes were imaged using a CCD camera (Bio-Rad) and analyzed in Image Lab.

### Effect of glucolipotoxicity on IRIS-1/2 expression in SH-SY5Y cells

Cells were seeded in 12-well plates (2 × 10^5^ cells/ well) or 24-well plates (1.5 × 10^5^ cells/ well) for IRIS-1 and IRIS-2 analysis, respectively. The cells were then treated for 48 h with Opti-MEM medium supplemented with various combinations of 25 mM glucose, 200 µM palmitic acid, 50 or 100 ng/ ml TNF-α ImmunoTools), and 100 ng/ ml IFN-γ (ImmunoTools). Medium was replaced after 24 h. The control wells were untreated, containing Opti-MEM medium only. For the Western blot, the antibodies used for IRIS-1 was Capra, custom made #614.610 or #613.610, 1:1000 in Quench, with the goat-anti-rabbit Abcam/Dako 1:5000. For IRIS-2 the Capra, custom made #612.609 antibody was used, 1:1000 in Quench with Dako goat-anti-rabbit 1:5000. For the loading control anti-β-tubulin, 1:25000 in Quench (Abcam #ab6046) was used, with the Dako goat-anti-rabbit 1:25000.

### qRT-PCR

RNA was extracted using RNeasy plus Mini kit (Qiagen) and cDNA was synthesized using oligo-dT primers and Superscript IV (Invitrogen). Quantitative PCR was performed with specific TaqMan probes (Applied Biosystem) and Viia7 Real-Time PCR system (Thermo Fisher). Expression levels of CD59, CDK5, microtubule-associated protein tau (MAPT), GSK3β, and ADAM17 (Thermo Scientific) were calculated after normalization with the mean of the housekeeping gene GAPDH. 2^-ΔΔCt^ method was used to determine the gene expression differences among the groups. For the standard PCR, the gels were imaged directly after electrophoresis using a CCD camera (Bio-Rad). The band intensities were then quantified from the ratio of IRIS-1/2 and CD59 to GAPDH in Image Lab (Bio-Rad).

### Noradrenaline secretion ELISA

SH-SY5Y cells were seeded on a 24-well plate. Knockdown of CD59, IRIS-1 and IRIS-2 was performed by incubation with siRNA targeting CD59, 300 nM (Ambion #4392422) and 1.5 µl lipofectamine RNAiMax (Invitrogen) for 72 h. Non-targeting negative control siRNA (Ambion, #4390843) was used as a negative control. After that time the cells were washed 1x with PBS and incubated for 30 min. at 37 °C with HEPES-buffered saline (HBS) (0,44 mM KH_2_PO_4,_ 1,2 mM MgCl_2,_ 2 mM CaCl_2,_ 4,2 mM NaHCO_3,_ 20 mM HEPES, 137 mM NaCl, 5 mM KCl, 5 mM glucose, 0.2 mM pargyline, 0.2 mM ascorbic acid, pH 7,4). Noradrenaline secretion was measured by incubating the cells for 30 min with HBS containing 5 mmol/L KCl (basal release), or with HBS containing 100 mmol/L KCl (evoked release). The supernatants were collected. Cells were washed 3x with ice-cold PBS, and lysed with a RIPA buffer containing protease and phosphatase inhibitors. The total protein content was measured using BCA kit (Thermo Scientific). Further, the noradrenaline level in the supernatant was determined and the cell extracts were used for total protein quantitation. The noradrenaline ELISA was performed according to the manufacturer instructions (EKN47439, 96 tests). Additionally, the CD59, IRIS-1 and IRIS-2 knockdown efficiency was verified by Western Blotting. The antibodies used were Capra #614.610 or #613.610 custom made antibody for IRIS-1, 1:1000 in EveryBlot/Quench with secondary Abcam/Dako 1:5000 goat-anti-rabbit. For IRIS-2 the Capra #612.609 custom made antibodies were used 1:1000 in Quench, with Dako goat-anti-rabbit 1:5000. For CD59 the BRICK 229 (IBGRL research #9409P) antibody was used, 1:2000 in 5% milk in immunowash, with Dako goat-anti-mouse 1:5000 as secondary antibody. For the loading control, anti-β-tubulin (Abcam #Ab6046) was used 1:25000/1:10000 in Quench with Dako goat-anti-rabbit 1:25000/1:10000.

### Effects of CD59 knockdown on p-tau and Cdk5 expression

Knockdown of CD59 was performed and its efficiency verified in the same way as described in the “Noradrenaline secretion assay” methods section. Western blotting was used to analyse the effects of CD59 knockdown on p-tau and Cdk5 expression. Membranes were probed with the following antibodies: anti-Cdk5 (1:1000, Abcam #2506) in Quench buffer, non-reduced conditions, Dako goat-anti-rabbit 1:2000, anti-Tau (1:1000, Invitrogen #AHB0042) in 5% BSA with Dako goat-anti-mouse 1:2000, AT8 (Ser202/ Thr205, 1:1000, Invitrogen #MN1020) in 5% BSA, with Dako goat-anti-mouse 1:2000, and anti-GAPDH (Abcam #Ab8245) 1:10000 in Quench buffer with Dako goat-anti-mouse 1:10000.

### Subcellular fractionation

SH-SY5Y cells were seeded on and harvested from a petri dish by trypsinization (5 × 10^6^ cells). Cells were then fractionated using MEM-PER Plus Kit (Thermo Scientific, #89842) according to the manufacturer’s instructions and analyzed by Western Blotting. The antibodies used for the western blot were anti-VAMP2 1:1000 (Synaptic Systems #104211) in 5% Milk, with Dako goat-anti-mouse 1:5000 as secondary, anti-PDI (Enzo life sciences #ADI-SPA-891-D) 1:100000 in EveryBlot, with Dako goat-anti-mouse 1:5000, anti-β-tubulin, 1:25000 in Quench (Abcam #Ab6046) with Dako goat-anti-rabbit 1:25000. For IRIS-1 the Capra custom made antibodies #613.610 or 614.610 were used, 1:1000 in Quench, with Abcam goat-anti-rabbit 1:5000. For IRIS-2 Capra custom made antibody #612.609 was used in 1:1000 in Quench, with Dako goat-anti-rabbit 1:5000.

### Proximity ligation assay (PLA)

PLA was carried out according to the manufacturer’s instructions (NaveniFlex Cell MR Red #NC.MR.100) to detect and quantify protein-protein interactions [17]. In short, two primary antibodies bind to their target epitopes, located on a single protein or two nearby proteins. Antibodies used: VAMP2 (1:100, Synaptic Systems #104211, mouse monoclonal) or VAMP2 (1:100, Synaptic Systems #104008, rabbit monoclonal. Used depending on antibodies pair, as the two different species should be used, ex. mouse-rabbit), IRIS-1/ 2 antibodies (1:100, Capra, custom made #613.610, #612.609 respectively), SNAP-25 (Abcam, #EPR3275), Syntaxin 1 (1:100, Synaptic Systems #110011) and incubated overnight in 4 °C. Further, Navenibodies (antibodies conjugated to proprietary oligonucleotide arms), which bind to their respective primary antibodies were added for 1 h, 37 °C. If the Navenibodies are in close proximity, the attached oligos can generate a DNA circle, which via addition of polymerase amplifies (rolling circle amplification process) generating fluorescent puncta (fluorescent probes are bound to the amplified DNA). The high signal-to-noise enables the detection of separate proximity events, allowing for a resolution down to a single protein-protein interaction. The interactions were then visualized using Carl Zeiss 800 confocal microscopy, 10–20 Z-stacks taken per image, merged using Zeiss software, and quantified using ImageJ program (functions: nuclei count, analyze particles with threshold set up on negative control: one antibody used).

### Quantification and statistical analysis

All experiments were performed in at least three independent repeats. All western blots show a representative of at least three independent repeats. The mean differences between groups that have been split into two independent variables were compared with 2-way ANOVA. Student’s t-test was used in case of two independent group comparisons, and 1-way ANOVA when three or more independent groups were compared. Normal distribution of data was verified with histogram and Q-Q plot, whereas the standard deviation between the groups was visually inspected with scatter plot. All statistical analyses were performed using GraphPad Prism 10, or R 4.3.3. Values are expressed as a mean ± SD. Statistical tests applied in each experiment are indicated in the figure legends. Bonferroni and Dunnett’s tests were used in case of multiple comparisons. All the experiments except brain samples immunostaining were repeated at least three times. In all figures, *p < 0.05, **p < 0.01 and ***p < 0.001.

## Results

### IRIS-1 and IRIS-2 are expressed in human neurons and astrocytes

**Fig. 1:**
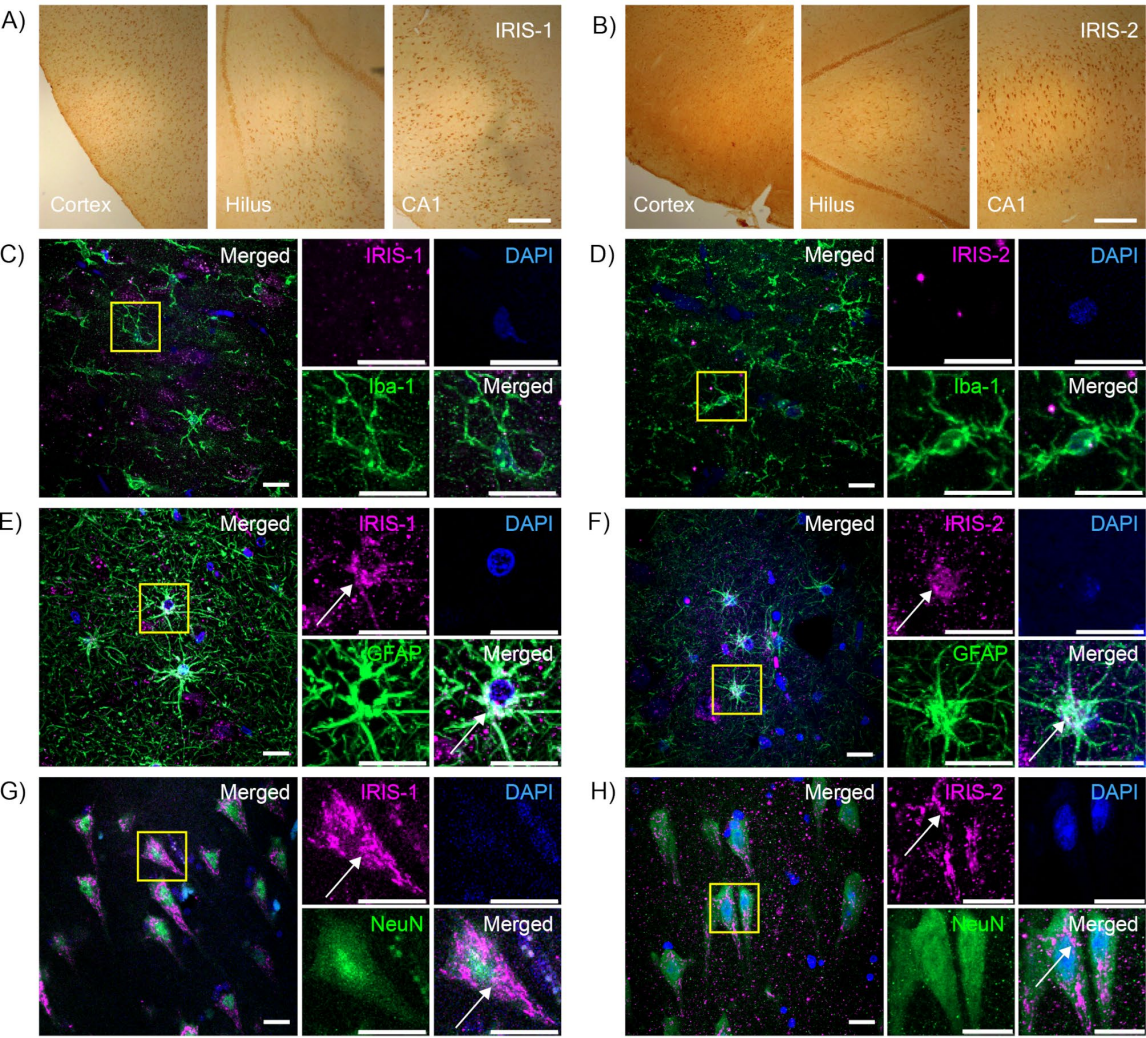
Images of hippocampus and entorhinal cortex from non-demented controls immunohistochemically stained against IRIS-1 (**A**) and IRIS-2 (**B**) show specific immunoreactivity in both cortex and the hippocampal subregions hilus and Cornu Ammonis 1 (CA1) Scale Bar = 400 μm. Images in (**C-H**) show representative images of hippocampal sections immunostained against IRIS-1 or IRIS-2 together with the microglia marker Iba-1, the astrocytic marker GFAP and the neuronal marker NeuN. Image in (**C** and **D**) shows a staining against IRIS-1 and IRIS-2 (purple in **C** and **D**, respectively) together with Iba-1 (green) and the nuclei staining DAPI (blue). Zoomed-in images of microglias in the yellow squares are shown to the right in (**C** and **D**). The images revealed no or very little colocalization between IRIS-1 and IRIS-2 and Iba-1. Images in (**E** and **F**) demonstrate GFAP positive astrocytes (green) co-stained against IRIS-1 and IRIS-2 (purple) and DAPI (blue). Zoomed-in images of astrocytes in the yellow squares are found to the right. The staining showed a moderate degree of colocalization between GFAP and IRIS-1 or IRIS-2 in astrocytes (indicated with arrows). Colocalization was also observed between IRIS-1 and IRIS-2 and the neuronal marker NeuN (visible as white puncta). Images, and zoomed-in neurons within yellow squares in (**G** and **H**) show localization of IRIS-1 and IRIS-2 (purple) within NeuN positive neurons (green) (indicated with arrows). The thickness of the Z-stack varies between 19.8 and 39.6 μm. Scale Bar = 20 μm

To investigate the presence of IRIS-1 and IRIS-2 in the human brain, we immunohistochemically stained the hippocampus and entorhinal cortex from NC (Group 1A) with specific antibodies against the two IRIS isoforms. The staining revealed a widespread expression of both IRIS-1 and IRIS-2 in cortex and hippocampal subregions including the hilus and the CA1/subiculum (Fig. 1A, B), CA2 and CA3. To further study the cellular localization of IRIS-1 and 2 in the human brain, we focused on three major cell populations: microglia, astrocytes, and neurons. Microglia, macrophage-like cells in the central nervous system (CNS), constitute 1–16% of the total cell population in the adult human brain, and are involved in phagocytosis of cellular debris and apoptotic cells, and control of neuronal excitability [18]. Our immunofluorescent staining of brain sections did not provide evidence for microglial expression of IRIS-1 and IRIS-2 as only very few or no IRIS-1 or -2 puncta was found within Iba-1 positive microglia (Fig. 1C, D). Astrocytes, comprising 20–40% of all glial cells, regulate blood-brain barrier permeability and support neuronal energy needs through the astrocyte-neuron lactate shuttle. They also modulate neuronal excitability by influencing Ca^2+^ levels and releasing gliotransmitters [19]. Our immunofluorescent staining revealed some colocalization between IRIS-1 and 2 and the astrocyte marker GFAP, evident as white puncta (Fig. 1E, F). Examples from the remaining analyzed cases are found in (Supplementary Fig. 1). The highest expression levels of IRIS-1 and 2 were found in the cell bodies of NeuN positive neurons localized within the CA1 region of the hippocampus (Fig. 1G, H).

### IRIS-1 and IRIS-2 are endogenously expressed in SH-SY5Y cells, and their expression increases in SH-SY5Y cells differentiated into mature neurons

**Fig. 2:**
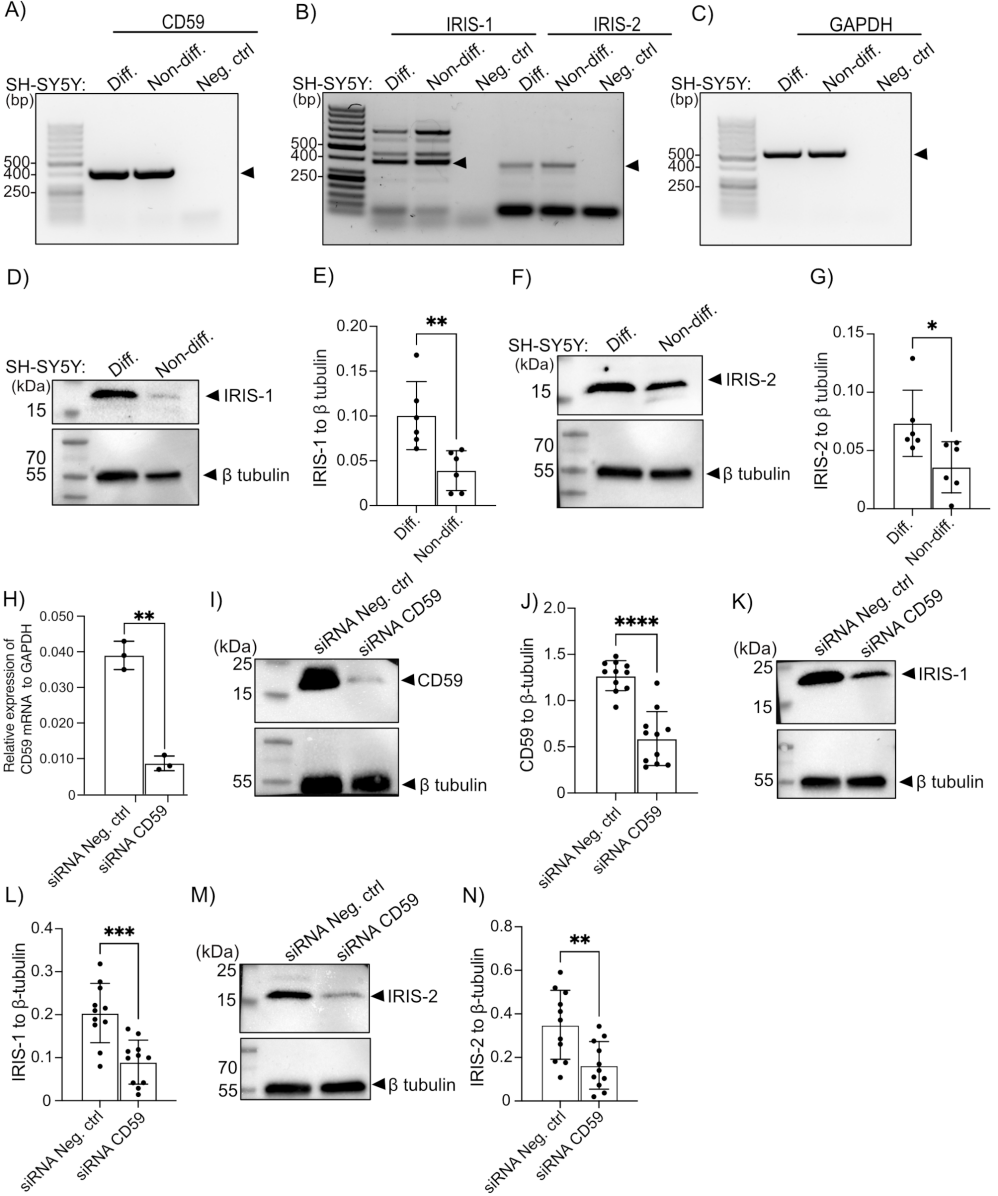
(**A**) Expression of canonical CD59 and (**B**) its isoforms: IRIS-1, and IRIS-2 in human neuroblastoma cells line (SH-SY5Y) undifferentiated and differentiated into mature neurons with BDNF and retinoic acid analyzed by semi-quantitative RT-PCR (*n* = 3). The negative control is the reaction mix, without template (no cDNA). (**C**) GAPDH served as a control. (**D**) Western blot analysis of IRIS-1 protein level in undifferentiated vs. differentiated SH-SY5Y cells, showing increased expression post-differentiation (*n* = 6). β tubulin was used as a loading control. The quantification is shown in (**E**). (**F**) Similarly, IRIS-2 protein levels were higher in differentiated cells, as shown by Western blotting (*n* = 6). Quantification is shown in (**G**). (**H**) CD59 expression in SH-SY5Y cells was measured by QRT-PCR to verify the mRNA level of CD59 knockdown (*n* = 3). Non-targeting negative control siRNA (Ambion, #4390843) was used as a negative control. GAPDH was used as a reference gene. (**I**) Western blot for CD59 protein to confirm knockdown efficiency for repeats from both the noradrenaline secretion assay- and p-tau/Cdk5 expression experiments, with β-tubulin as a loading control (*n* = 11). Quantification shown in (**J**). (**K**, **M**) Western blotting assessed IRIS-1 and IRIS-2 protein levels post-knockdown, respectively (*n* = 11, each). Quantifications are shown in (**L** and **N**). Statistics (in **E**, **G**, **H**, **J**, **L**, and **N**): two-tailed student T-test. Error bars indicate SD

To investigate the functional relevance of IRIS-1 and IRIS-2 in human neurons, we utilized the SH-SY5Y human neuroblastoma cell line model, a common choice for studying neuronal function and neurodegenerative disorders [20]. Treatment of the SH-SY5Y cells with BDNF and ATRA differentiates the cells into mature, neuronal phenotype [20]. SH-SY5Y cells differentiate primarily to a cholinergic neuron phenotype in response to ATRA treatment, as evidenced by increased choline acetyltransferase activity and vesicular monoamine transporter (VMAT) expression [20]. We detected a relatively high mRNA expression of canonical CD59 (Fig. 2A) as well as IRIS-1 and 2 (Fig. 2B, C) in differentiated and undifferentiated SH-SY5Y cells. Subsequently, we confirmed the presence of IRIS-1 and 2 at the protein level in undifferentiated SH-SY5Y cells. Interestingly, we observed a significant increase in the protein expression levels of IRIS-1 and IRIS-2 in SH-SY5Y cells differentiated into mature neurons using BDNF and ATRA, as shown in (Fig. 2D, quantification in **E**) for IRIS-1 and (Fig. 2F, quantification in **G**) for IRIS-2. To assess the efficiency of differentiation of the SH-SY5Y cells, the RNA from undifferentiated and differentiated cells was isolated and verified for the increase of expression in mature neuronal markers, including synaptophysin (Supplementary Fig. 2A), enolase 2 (Supplementary Fig. 2B), microtubule-associated protein 2 (MAP2) (Supplementary Fig. 2C), and secretogranin II (Supplementary Fig. 2D).

### IRIS-1 and IRIS-2 interact with secretory vesicles, and their silencing alongside canonical CD59 affects the SNARE complex formation, leading to a reduction in noradrenaline secretion

**Fig. 3:**
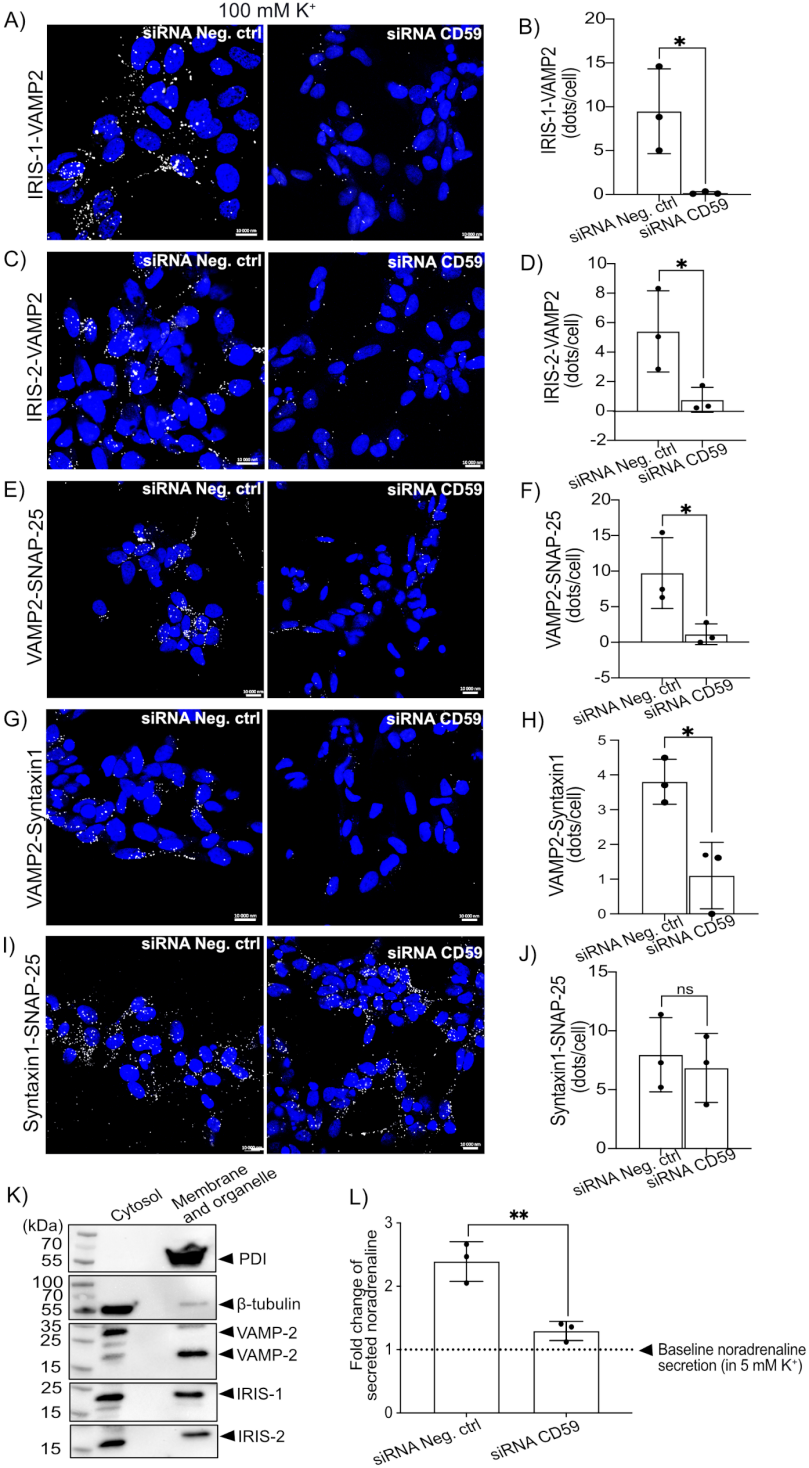
Proximity ligation assay was used to assess interactions (represented by white dots) between IRIS-1 with VAMP2 (**A** with quantification in **B**) and IRIS-2 with VAMP2 (**C** with quantification in **D**) under high potassium stimulation. Proximity ligation assay was used to assess the SNARE complex formation under high potassium stimulation in SH-SY5Y cells treated with siRNA negative control and siRNA targeting CD59, IRIS-1, and 2. The following complexes were assessed: VAMP2 and SNAP-25 (**E** with quantification in **F**), VAMP2 and Syntaxin1 (**G** with quantification in **H**), Syntaxin 1 and SNAP-25 (**I** with quantification in **J**). *n* = 3, biological repeats in (**A-J**). (**K**) Subcellular fractionation of SH-SY5Y cells analyzed through western blot showing that IRIS-1 and 2 localizes within the same cellular compartments as VAMP2. The purity of fractions were assessed with specific antibodies form disulfide isomerase (PDI), specific for membranes, and β-tubulin, enriched in cytosolic fraction. *n* = 3. (**L**) Noradrenaline secretion from SH-SY5Y cells with or without CD59, IRIS-1, and 2 knockdown. Data are represented as a fold change of secreted noradrenaline in cells stimulated with 100 mM potassium (evoked noradrenaline release) compared to cells stimulated with 5 mM potassium (baseline noradrenaline secretion, represented as a dotted line). The amount of secreted noradrenaline in 5 mM and 100 mM potassium was normalized to total protein content, representing the number of cells. Statistics (in **B**, **D**, **F**, **H**, **J**, and **L**): two-tailed student T-test. The thickness of the Z-stack varies between 19.8 and 39.6 μm

To study the functional outcome of IRIS-1, and IRIS-2 expression in SH-SY5Y cells, we performed the knockdown of all the CD59 splice variants as no specific siRNAs are available that can distinguish between these three transcripts. Real-time PCR showed a marked reduction of mRNA transcript for canonical CD59 when siRNA against CD59 was used, compared to non-targeting control (Fig. 2H). Efficient protein-level downregulation of CD59 expression was also detected in **both the noradrenaline secretion assay- and p-tau/Cdk5 expression experiments (**Fig. 2I, quantification in **J**), consistent with mRNA results. We also detected significant downregulation of both CD59-IRIS protein isoforms, as shown in (Fig. 2K, quantification in **L**) for IRIS-1 and (Fig. 2M, quantification in **N**) for IRIS-2. Since the release of neurotransmitters involves the formation of the SNARE protein complex (consisting of vesicle-bound VAMP2, and plasma membrane-bound Syntaxin1, and SNAP-25), to induce a vesicle-plasma membrane fusion [21], we next investigated whether IRIS-1 and 2 interact with secretory vesicles in SH-SY5Y cells by binding to vesicle-associated VAMP2. Before assessing the binding, the cells were stimulated with high potassium concentrations, mimicking the evoked secretory state. Using PLA, we detected an interaction between VAMP2 and both IRIS-1 (Fig. 3A, quantification in **B**) as well as IRIS-2 (Fig. 3C, quantification in **D**), implicating their association with the secretory vesicles. We also tested whether IRIS-1 and 2 could interact with other small intracellular vesicles, including endosomes, which we stained with early endosomal antigen 1 (EEA1). However, we did not detect any colocalization between IRIS-1 and 2 and EEA1, as shown by immunofluorescence in (Supplementary Fig. 3), suggesting that IRIS-1 and 2 are preferably localized to secretory vesicles. Further, we detected that the silencing of IRIS-1 and 2 significantly decreased the SNARE complex assembly (in cells stimulated with evoked potassium concentrations) between VAMP2 and SNAP-25 (Fig. 3E, quantification in **F**), and VAMP2 and Syntaxin1 (Fig. 3G, quantification in **H**). As expected, no differences in SNARE complex formation between Syntaxin 1 and SNAP-25 were detected, which are both localized together at the plasma membrane (Fig. 3I, quantification in **J**). Furthermore, when fractionating SH-SY5Y cells, IRIS-1 and 2 were found in the same cellular compartments as VAMP2 (Fig. 3K). SH-SY5Y cells contain proteins needed for regulated secretion and functional dense core vesicles capable of releasing noradrenaline evoked by K^+^ depolarization [22], thus providing a suitable model for studying exocytosis. We detected significantly decreased levels of secreted noradrenaline from cells in which all the CD59 splice variants were silenced (Fig. 3L). The data were represented as fold change in the amounts of secreted noradrenaline in evoked conditions, 100 mM K^+^, compared to baseline noradrenaline secreted in 5 mM K^+^, represented with a dotted line. CD59 mRNA and protein-level silencing efficiency is shown in (Supplementary Fig. 4).

### Expression of IRIS-1 and IRIS-2 is significantly reduced in neurons from AD and T2D individuals and in the late stages of tauopathy

**Fig. 4:**
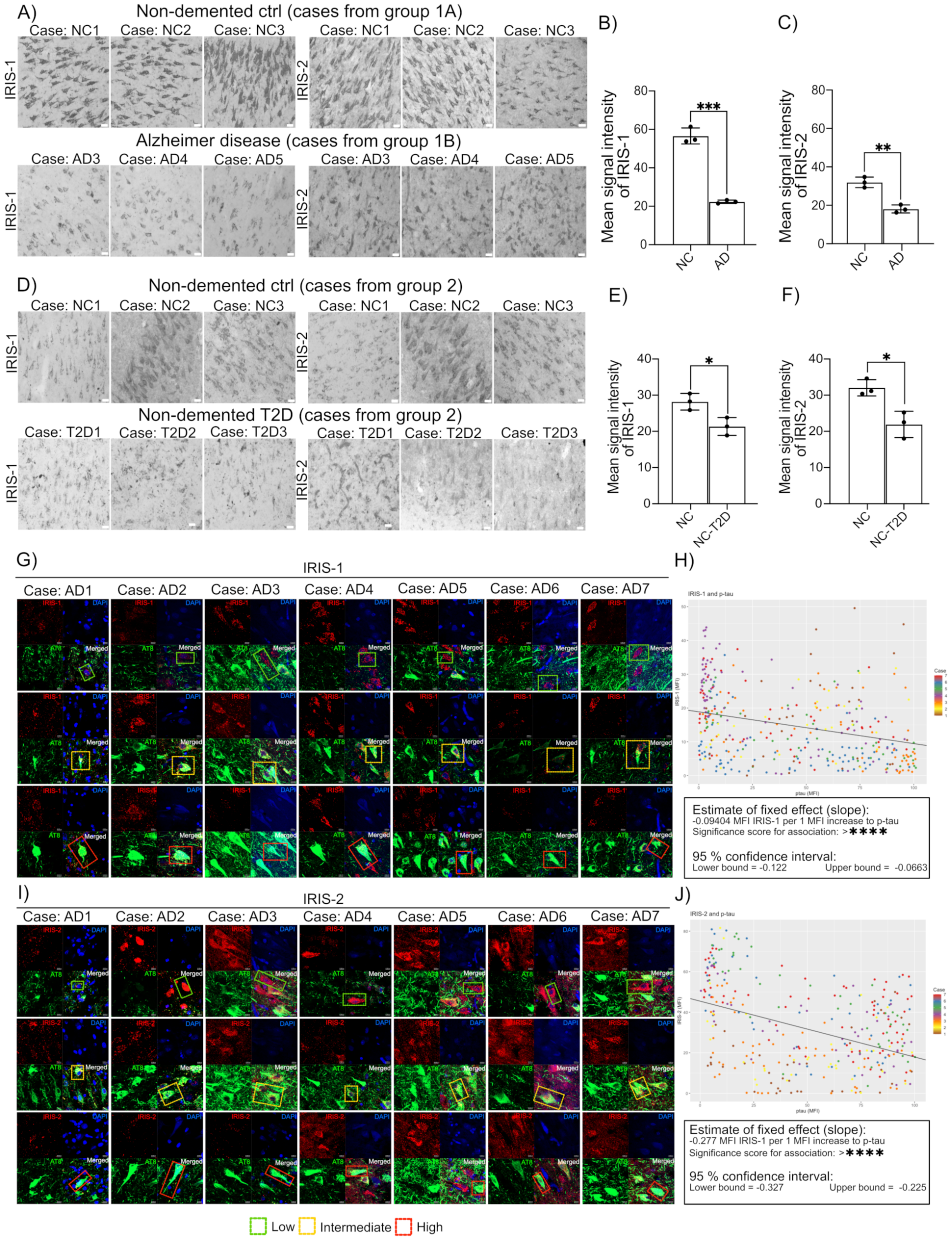
Images in (**A**) show representative images of human hippocampal sections from three AD cases and three NC (Group 1 A, 1B) immunohistochemically stained against IRIS-1 and IRIS-2. The graphs in (**B**, **C**) demonstrate significantly lowered IRIS-1 of IRIS-2 immunoreactivity in the CA1 region of AD compared to NC (each dot represents the mean value of 6 images with 10–12 neurons used for quantification from every image. Each group consists of *n* = 3 cases). In (**D**) representative images of the CA1 region in three T2D cases and three NC are shown (Group 2). The graph in (**E**, **F**) shows the significantly lowered IRIS-1 of IRIS-2 immunoreactivity in T2D cases compared to NC (each dot represents the mean value of 6 images with 10–12 neurons used for quantification from every image. Each group consists of *n* = 3 cases). Statistical analysis of changes between NC vs. AD and NC vs. T2D was performed using two-tailed student T-tests, with error bars indicating standard deviation (SD). Of note, the difference in immunoreactivity seen in (**A** and **D**) is due to the different postfixation methods used after autopsy, where tissue from cases in Group 1 A and B (**A**) were immersion fixed in PFA directly after autopsy, while tissue from cases in Group 2 (**C**) was first snap frozen, followed by immersion fixation in PFA. Scale bars = 50 μm. Images in (**G**, **I**) show representative immunofluorescence stainings against IRIS-1 and IRIS-2 (red), tau phosphorylated at Ser 202/ Thr205 (AT8) (green) and DAPI (blue) of sections from the AD cases included in Group B (*n* = 7 AD cases). IRIS-1 (**G**) and IRIS-2 (**I**) positive neurons in the CA1 regions was divided into low, intermediate or high AT8 immunoreactivity, where low represents healthy neurons (very low or no AT8 staining), and high represents neurons with high amount of AT8 immunoreactivity (each representative neuron is indicated with dotted rectangles, green for low, yellow for intermediate and red for high, *n* ≥ 30 neurons per case and *n* ≥ 10 neurons per AT8 immunoreactivity category was analyzed). The thickness of the Z-stack varies between 19.8 and 39.6 μm. Scale bars = 10 μm. Dotplots for all neurons analysed were included and color coded by case number, with the estimate of the fixed effect as the slope, shown in (**H**) for IRIS-1 and (**J**) for IRIS-2

Depletion of SNAP-25 or Syntaxin1 induces neurodegeneration, and neuronal death respectively [23, 24] suggesting SNARE protein involvement in the pathology of neurodegenerative diseases. Additionally, brain tissues from AD patients show neurotransmission defects, due to impairment of neurotransmitter exocytosis [24]. Using specific antibodies, we determined expression of IRIS-1 and 2 in human brain pathologies. We detected significantly reduced levels of neuronal IRIS-1 and 2 in hippocampus of AD patients, compared to healthy controls (Fig. 4A, quantification in **B** and **C**). Compared with the general population, patients with Type 2 diabetes (T2D) suffer a 50–150% increased risk of developing dementia, particularly AD [25]. We previously reported that glucotoxicity causes a decreased expression of IRIS-1 and 2 in human pancreatic islets [10]. We now detected significant downregulation of IRIS-1 and 2 expressions in CA1 neurons in non-demented T2D individuals, compared to healthy controls (Fig. 4D, quantification in **E** and **F**). AD consists of two main neuropathological lesions: amyloid plaques and neurofibrillary tangles of hyper-phosphorylated tau protein [26]. We observed a high expression of IRIS-1 and 2 in healthy neurons, similarly to neurons with early stages of tau-pathologies (detected with AT8 antibody specific for tau phosphorylated at Ser 202 and Thr 205), and almost complete loss of IRIS-1 and 2 expressions in neurons with neurofibrillary tangles, as shown in (Fig. 4G) for IRIS-1, and (Fig. 4I) for IRIS-2. The mixed model statistical analysis showed a significant (*p* < 0.0001) although weak negative association between IRIS-1 and p-tau, with an estimated decrease of -0.09404 mean fluorescent intensity (MFI) of IRIS-1 per 1 MFI of p-tau, 95% confidence interval (CI) (lower bound: -0.122, upper bound: -0.0663), all neurons shown in (Fig. 4H), and a significant (*p* < 0.0001) somewhat stronger negative association for IRIS-2, with an estimated decrease of -0.276 MFI of IRIS-2 per 1 MFI of p-tau, 95% CI (lower bound: -0.327, upper bound: -0.225), all neurons shown in (Fig. 4J). The model assumptions were assessed and fulfilled. This suggests that the expression of IRIS-1 and 2, and thereby the release of neurotransmitter (as indicated by our cell culture studies), reduces along with the accumulation of p-tau and NFT formation. In case of prolonged neuronal assault and progression from early tau pathologies to neurofibrillary tangles, the expression of IRIS-1 and 2 decreases, which can be an additional factor contributing to neurotransmission failure.

### Expression of IRIS-1 and IRIS-2 are significantly downregulated by glucotoxicity and proinflammatory cytokines

**Fig. 5:**
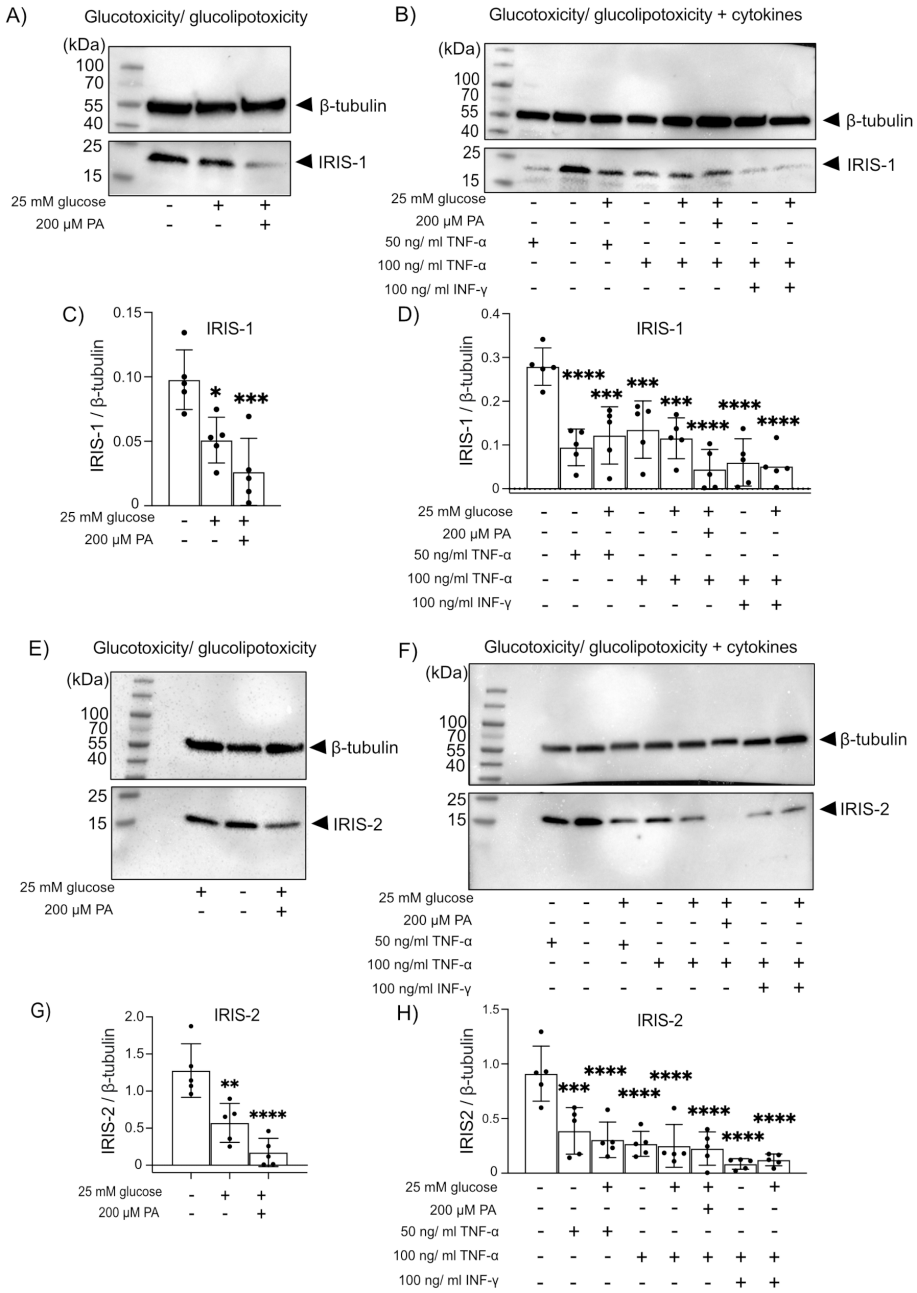
(**A**) Western blots showing the impact of glucose and glucolipotoxicity treatment (induced by with glucose and palmitic acid for 48 h) on IRIS-1 protein expression, and (**B**) the effect of gluco-lipo-toxicity in combination with pro-inflammatory cytokines (TNF-α, IFN-ɣ) on IRIS-1 expression, in neuroblastoma cell line (SH-SY5Y) to induce conditions mimicking AD and T2D. Quantifications are shown in (**C**) and (**D**) respectively (*n* = 5). (**E**) Western blots showing the effect of glucose and glucolipotoxicity on IRIS-2 expression, and (**F**) effects of glucolipotoxicity and pro-inflammatory cytokine addition on IRIS-2 expression, with quantifications shown in (**G**) and (**H**), respectively (*n* = 5). Graphs represent the ratio of band intensity for IRIS-1 or 2 to β-tubulin. Statistical analyses for panels (**C**, **D**, **G** and **H**) were performed using one-way ANOVA. Error bars indicate standard deviation (SD). Each group was compared to the untreated group (first bar) to determine the statistical significance

In diabetic patients, inadequate glucose control is linked to decreased cognitive function [27], as high serum glycated hemoglobin (HbA1c) levels are associated with decreased memory, learning, and complex psychomotor performance [27]. Additionally, cytokines, which in a healthy brain are key players in maintaining CNS homeostasis, turn aberrant during AD development, which underlies neuroinflammation, a central feature of AD, in which over-activated microglia increase the production of proinflammatory cytokines driving neuronal cells toward apoptotic decline [28]. We, therefore, checked whether prolonged elevated glucose and palmitic acid concentrations (mimicking glucotoxicity and lipotoxicity), both present in T2D individuals [29] or proinflammatory cytokines, including TNF-α and IFN-ɣ (detected in AD brains [29] or a combination of both factors would affect the expression of IRIS-1 and 2 isoforms in neurons. The expression of both IRIS-1 and IRIS-2 significantly decreased in SH-SY5Y cells during all treatments. A decrease in IRIS-1 level upon gluco- and glucolipo-toxicity is shown in (Fig. 5A, quantification in **C**), and with additional cytokines treatment in (Fig. 5B, quantification in **D**). Similarly, decreases in IRIS-2 were also seen upon gluco- and glucolipo-toxicity (Fig. 5E, quantification in **G**) and with additional cytokines treatment (Fig. 5F, quantification in **H**). The greatest decrease of IRIS-1 and IRIS-2 expression is seen in conditions combining high concentrations of TNF-α, IFN-ɣ, glucose and palmitic acid. Changes in mRNA expression levels for IRIS-1, 2 and CD59 were measured by semi-quantitative PCR performed on mRNA samples, as no specific probes with TaqMan detection system exist for IRIS-1 and 2. Semi-quantitative PCR (with GAPDH as internal reference) also confirmed significant downregulation of IRIS-1 and IRIS-2 mRNA expression levels upon treatment with elevated glucose, palmitic acid, and proinflammatory cytokine TNF-α, as shown in (Supplementary Fig. 5A, quantification in S5D) for IRIS-1, and in (Supplementary Fig. 5B, quantification in S5E) for IRIS-2 transcript. Interestingly no difference in mRNA expression of canonical CD59 was observed, as shown in (Supplementary Fig. 5C, quantification in S5F), confirmed by no differences in CD59 protein level expression shown in (Supplementary Fig. 5G, quantification in S5H).

### Silencing IRIS-1 and IRIS-2 in SH-SY5Y cells leads to higher expression of Cdk5 and accumulation of phosphorylated tau in these cells

**Fig. 6:**
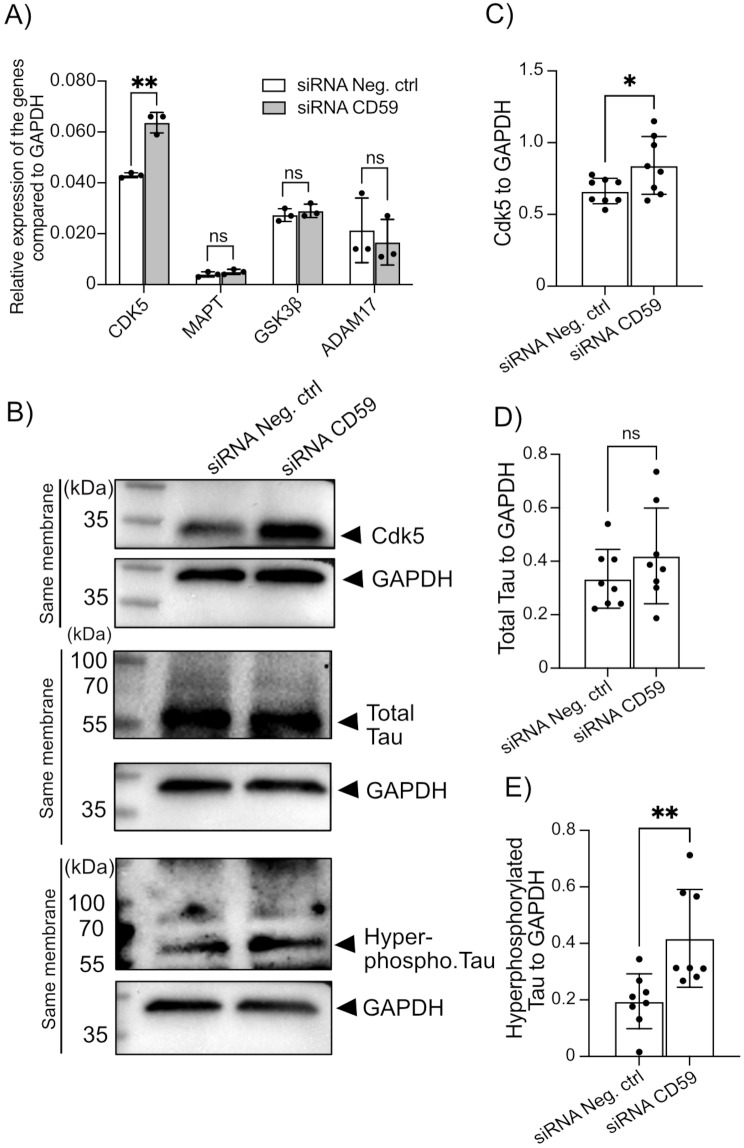
(**A**) QRT-PCR analysis of mRNA levels for CDK5, MAPT, GSK3β, and ADAM17 in SH-SY5Y cells with and without CD59, IRIS-1, and IRIS-2 knockdown, using GAPDH as a reference gene (*n* = 3). (**B**) Western blotting assessments of separate representative repeats for Cdk5, total tau, and phosphorylated tau (AT8 antibody targeting phosphorylation at Ser 202, Thr 205) protein levels, respectively, in SH-SY5Y cells with and without CD59, IRIS-1, and IRIS-2 knockdown. Each representative membrane is grouped with its loading control (GAPDH) as indicated by the lines. Quantifications for each protein, based on eight biological repeats, are presented in (**C**, **D** and **E**), respectively. Statistical analyses for panel (**A**) were performed using two-way ANOVA and for panels (**C**, **D** and **E**) using two-tailed student T-test, with error bars indicating standard deviation (SD)

Tau pathology, characterized by increased phosphorylation and aggregation of tau protein, is closely related to neuronal loss in AD [30]. Cyclin-dependent kinase 5 (Cdk5) is a main kinase involved in abnormal tau phosphorylation in AD brains [31]. We determined that the mRNA level of expression of CDK5 gene is higher in cells in which CD59, IRIS-1, and IRIS-2 were silenced by siRNA (Fig. 6A). It is established that increased expression of Cdk5 corresponds to its higher activity [32]. Indeed, we observed increased protein expression levels of Cdk5 in SH-SY5Y cells with IRIS-1 and 2 knockdown (Fig. 6B, quantification in **C**). We observed no differences in the mRNA expression level of another tau kinase, GSK3β (also known as TPKI) (Fig. 6A), as well as ADAM17, one of the metalloproteases involved in the cleavage of amyloid precursor protein (Fig. 6A). Reports showed that higher activity and expression of Cdk5 induces tau protein hyperphosphorylation [33]. Accordingly, while the mRNA levels (Fig. 6A) and total tau levels in cells remained unchanged (Fig. 6B, quantification in **D**), the levels of phosphorylated tau were significantly increased in cells with silenced CD59/ IRIS-1/ IRIS-2 (Fig. 6B, quantification in **E**). This suggests that IRIS-1 and 2 proteins may be involved in modulating Cdk5 expression and/ or activity, resulting in increased pathological phosphorylation of tau, exacerbating AD disease pathology.

## Discussion

The aim of this study was to investigate the cellular localization of CD59 isoforms IRIS-1 and 2 in the brain and explore their role in AD pathology. By performing immunostaining of postmortem human hippocampal tissue, we can for the first time, report expression of IRIS-1 and 2 in neurons. Besides neuronal expression, we also found an expression of IRIS-1 and 2 in astrocytes, but very little or none in microglia. This result could be explained by the fact that astrocytes express the same SNARE proteins isoforms as those present in neurons e.g. SNAP-25 or VAMP-2 [34]. The SNARE expression profile of microglia, however, differs from those of neurons and astrocytes. For example, microglia express SNAP-23 isoform, needed for their phagocytic function [34]. IRIS-1 and 2 were also detected in SH-SY5Y cells and, importantly, their expression increased when the cells were differentiated into mature neurons. This result strengthens the idea that IRIS proteins are involved in neurotransmitter release, as differentiation is required to form functional synapses in SH-SY5Y cells. The IRIS-1 and 2 in the SH-SY5Y cells were furthermore closely associated with VAMP2, supporting its implication in SNARE complex formation and indeed, silencing of all isoforms of CD59 including IRIS-1 and 2 led to a significant reduction in noradrenaline secretion. Altogether, this points toward an essential role of IRIS-1 and 2 in functional SNARE-dependent neurotransmitter release and neurotransmission. This is in line with our findings from pancreatic β-cells [8, 10], which also show a role for CD59 isoforms in SNARE complex formation and interactions with SNARE proteins, required for insulin granule exocytosis. As the IRIS isoforms appear to be retro-translocated from the ER lumen into the cytosol [9], it is predicted that this interaction occurs at the cytosolic face of secretory granules.

The importance of a functional SNARE complex for neurotransmission becomes evident when studying neurodegenerative diseases, such as AD or Parkinson’s disease. Expression of VAMP2, Syntaxin1, and SNAP-25 is reduced in human AD brain samples [24], and intracellular Aβ oligomers have been shown to bind to SNARE proteins and thereby inhibit the SNARE complex formation, blocking neurotransmission and exacerbating cognitive defects [35]. Our data are consistent with these findings, as they showed marked downregulation of IRIS-1 and 2 expressions in hippocampal neurons in AD and T2D cases, compared to controls. The results are also in line with the previously reported decrease in CD59 levels in AD patients [5], but the mechanism leading to the reduced IRIS-1 and 2 expression remains unknown. Since we previously reported that glucotoxicity causes a decreased expression of IRIS-1 and 2 in human pancreatic islets [10], it is plausible that glucotoxicity could be implicated in their reduction also in neurons. Importantly, epidemiological studies have shown an association between T2D and an increased risk of developing AD [25]. In addition, in diabetic patients, high HbA1c levels are associated with decreased memory and complex psychomotor performance, suggesting that inadequate glucose control may be associated with worsening cognitive function [27]. Recent literature further suggests that the mechanisms for the association between T2D and high AD risk revolves around glucotoxicity, and its downstream consequences like insulin resistance, impaired insulin receptor signaling, advanced glycation end products, or inflammation [36, 37]. From this perspective, it is interesting that we found a significant decrease in IRIS-1 and 2 expressions in SH-SY5Y cells upon prolonged glucose and palmitic acid exposure. Such glucolipotoxicity is known to induce insulin resistance and neurotoxicity in these cells [38, 39], where palmitate reduces insulin-induced signaling, and increases inflammatory cytokine expression, oxidative stress, and apoptosis. Similar glucolipotoxic conditions also caused downregulation of IRIS-1 and 2 in pancreatic β-cells, where they are involved in insulin granule exocytosis [10]. IRIS-1 and IRIS-2 are alternative splice products from the CD59 locus, which have a tissue-specific expression pattern [10], in comparison to the ubiquitous expression of the canonical CD59 isoform. While the regulation of IRIS-1 and IRIS-2 splicing is not currently understood, it is of interest that treatment of SH-SY5Y cells with palmitate led to downregulation of IRIS-1 and 2, but not canonical CD59, suggesting that the specific mechanism of IRIS-1 and 2 splicing was affected, without altering canonical CD59 promoter activity. Further investigation of this phenomenon could therefore improve understanding of specific IRIS-1 and 2 splicing regulation in secretory cells.

Tau is a member of the heat-stable microtubule-associated proteins (MAPs) family [40]. In physiological conditions, tau binds to axonal microtubules, facilitating microtubule assembly and stabilization, and contributing to the organization of the cytoskeleton, a process regulated by the phosphorylation of tau. Hyperphosphorylation of tau and downstream formation of NFTs is a hallmark of AD. The pyramidal neurons, for example those found in CA1, are particularly vulnerable, and the transition of a healthy pyramidal neuron into NFTs contributes to the neurodegeneration occurring in AD patients [41]. Interestingly, our immunostaining of AD hippocampal samples showed that IRIS-1 and 2 immunoreactivities were almost completely lost in NFTs. The association between p-tau and the two IRIS isoforms was also found in our experimental studies, where p-tau levels in SH-SY5Y cells increased after silencing IRIS-1 and 2. The underlying molecular pathway behind the association needs to be determined in detail, but our data suggests that Cdk5 is implicated, as its levels increased after IRIS isoforms silencing. In support, Cdk5 is a member of the Ser/ Thr cyclin-dependent kinase family and a main tau kinase known to be involved in abnormal phosphorylation in AD brains [33, 42]. The activation mechanism of Cdk5 is poorly understood, however, it is suggested that p35 could be the major activator of Cdk5. When neurons suffer from stress, a large influx of Ca^2+^ enters the neuronal cytoplasm, activating the calcium-dependent protease calpain. Calpain cleaves p35 to p25, and p25 binds the C-terminal Cdk5 activation domain [43]. It needs to be further determined whether the higher expression of Cdk5 seen in IRIS-1 and 2 silenced SH-SY5Y cells is driven by calpain and p35 cleavage mechanisms.

Neuroinflammatory processes accompany the accumulation of Aβ plaques and NFTs in the AD brain. The AD-related neuroinflammation is defined as a chronic activation of glial cells, primarily microglia and astrocytes, and subsequent release of pro-inflammatory cytokines and chemokines, surrounding senile plaques and affected neurons in the brain [44]. The release of pro-inflammatory cytokines, including TNF-α, IFN-ɣ or IL-1β, leads to synaptic dysfunction, neuronal death and inhibition of neurogenesis [44]. Additionally, the complement system can be activated, promoting the phagocytic function of microglia, which might result in inappropriate pruning of synapses. All these events create a vicious circle inducing further inflammation, recruiting immune cells, and increasing the production of reactive oxygen species (ROS), which can damage cellular components, leading to further neuronal injury. Our data demonstrated that prolonged exposure to pro-inflammatory cytokines, TNF-α and IFN-ɣ, significantly decreases the expression of IRIS-1 and 2 in SH-SY5Y cells. In view of this result, we speculate that neuroinflammation decreases the expression of IRIS-1 and 2, which in turn disturbs the SNARE complex formation, decreases neurotransmitter release, and causes further accumulation of hyperphosphorylated tau in neurons, leading to the progression of AD pathology.

One limitation of this study lies in the low number of cases included in the postmortem immunostaining study. This is due the fact that we have limited access to tissue post-fixed according to the specific protocols we use for immunostainings. We therefore encourage studies on larger cohorts to confirm the reduced presence of IRIS-1 and 2 in AD and T2D patients. However, the cohort size is sufficient to identify the cellular localization of IRIS-1 and 2. Additionally, by analyzing the presence of p-tau alongside IRIS-1 and IRIS-2 in over 300 neurons per isoform, we are confident that our analysis is robust enough to reveal potential associations between p-tau and these markers. Another limitation involves the interpretation of the results found in the postmortem study. Although the negative association between p-tau and IRIS-1 and 2 in the CA1 neurons indicate a relationship between the two variables, it is important to acknowledge that AD pathology contributes to a significant neuronal damage, and the found reduction of IRIS-1 and 2 might be a consequence of an AD-related neuronal damage, rather than an event triggering AD pathology. This “chicken and the egg” problem cannot be solved solely by analyzing postmortem samples, as they provide only a snapshot of events at time of death. Nevertheless, our cell culture studies demonstrating increased p-tau accumulation when all isoforms of CD59 are silenced support the idea that IRIS reduction, at least in vitro, is an event upstream to p-tau accumulation. Finally, it is important to note that cell culture studies never can replicate the complex biological events occuring in the living human brain. Therefore, our cell culture findings should be interpreted with caution and primarily viewed as a tool to explore and investigate isolated mechanisms in a controlled environment.

## Conclusions

In summary, our findings highlight the novel roles of IRIS-1 and IRIS-2 in the human brain, elucidating their critical functions in neurotransmitter secretion and their involvement in the pathologies of Alzheimer’s disease and type 2 diabetes. The observed association between the downregulation of these isoforms and neurodegenerative processes, coupled with their link to metabolic stress, underscores the potential of targeting IRIS-1 and IRIS-2 as a therapeutic strategy.

## Electronic supplementary material

Below is the link to the electronic supplementary material.

Supplementary Material 1

## Data Availability

No datasets were generated or analysed during the current study.

## Abbreviations

AD: Alzheimer’s disease
Aβ: Amyloid beta
NFTs: Neurofibrillary tangles
T2D: Type 2 diabetes
CNS: central nervous system
ER: Endoplasmic reticulum
GBS: Guillain–Barré syndrome
MAC: Membrane attack complex
SNARE: Soluble N-ethylmaleimide-sensitive factor attachments protein receptor
SYTs: Synaptotagmins
VAMP2: Vesicle associated membrane protein 2
SNAP25: Synaptosomal- associated protein 25
EEA1: Early endosomal antigen 1
VMAT: Vesicular monoamine transporter
IRIS-1, IRIS-2: Isoforms Rescuing Insulin Secretion 1 and 2
MAPT: Microtubule-associated protein tau
p-tau: Hyperphosphorylated tau protein
CDK5: Cyclin-dependent kinase 5
ADAM17: Metalloprotease 17
GSK3β: Glycogen synthase kinase 3 beta
TNF-α: Tumor necrosis factor alpha
INF-ɣ: Interferon-gamma
GAPDH: Glyceraldehyde-3-phosphate dehydrogenase
HbA1C: Hemoglobin A1C
PLA: Proximity ligation assay
KO: Knockout
NC: Non-demented controls
ATRA: All-trans retinoic acid
BDNF: Brain-derived neurotrophic factor
DMEM: Dulbecco’s Modified Eagle Medium
MFI: Mean fluorescent intensity
PFA: Paraformaldehyde
