# Supplementary material for: Neuronal CD59 isoforms IRIS-1 and IRIS-2 as regulators of neurotransmitter release with implications for Alzheimer’s disease
Source: Alzheimers Res Ther. 2025 Jan 7;17:11. doi: 10.1186/s13195-024-01660-z (PMC11705862; doi:10.1186/s13195-024-01660-z)
Supplement: Supplementary file 1 — Supplementary Material 1 [file 13195_2024_1660_MOESM1_ESM.pdf]

Supplementary figure 1

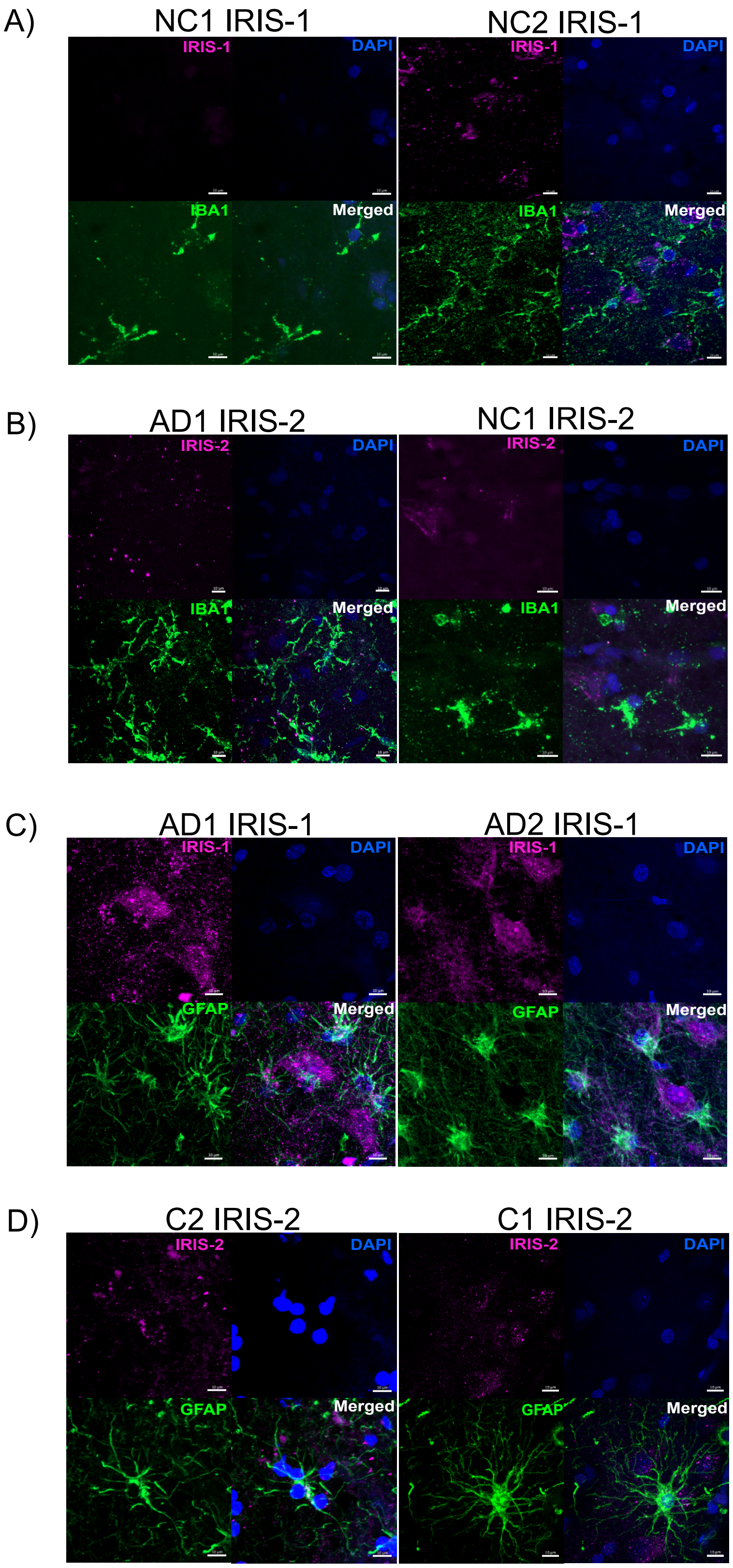

Figure S1: Immunofluorescent staining of hipocampal brain sections from cases NC1-2 and AD1-2 with three cases per costaining, using specific antibodies against IRIS-1 **(A),** IRIS-2 **(B)** (pink), and Iba1- marker of microglia (green) showed no or very little colocalization between IRIS-1, IRIS-2 and microglia. Immunofluorescent staining of the astrocytes using GFAP marker (green) showed moderate colocalisation with IRIS-1 (pink) visible as white puncta on **(C)**, and with IRIS-2 on **(D)**. Nuclei were stained with DAPI (blue).

Supplementary figure 2

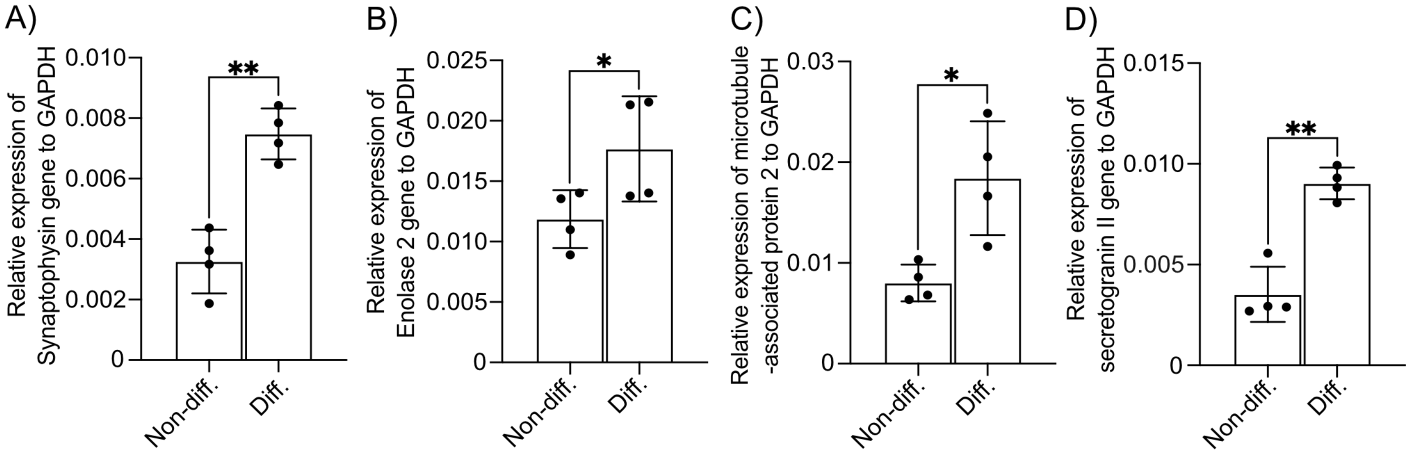

Figure S2: qRT-PCR analysis of mRNA expression level of mature neurons markers, such as synaptophysin (**A**), enolase 2 (**B**), microtubule-associated protein 2, MAP2 (**C**), and secretogranin II (**D**) in non-differentiated and differentiated into mature neurons (with retinoic acid and BDNF) SH-SY5Y cells. GAPDH was used as a internal reference gene. n= 4. Statistical analysis for all the panels was performed using student T-test, with error bars indicating standard deviations (SD).

Supplementary figure 3

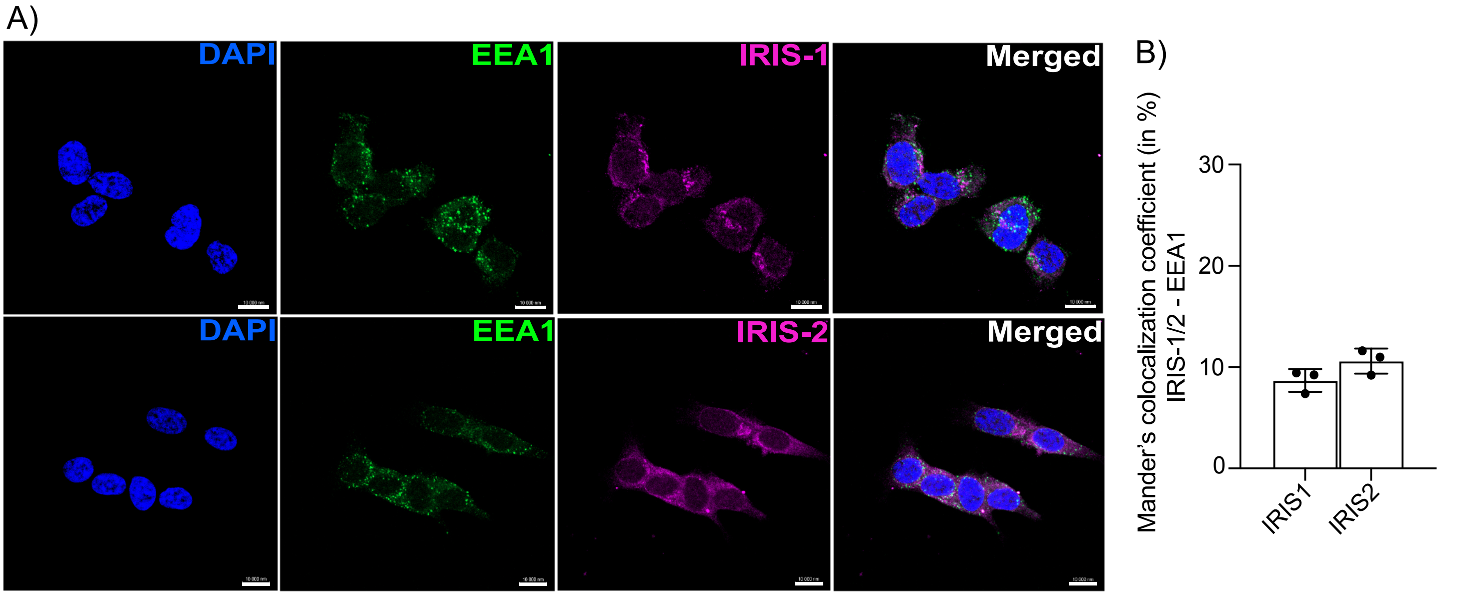

Figure S3: (**A**) Immunofluorescent staining of SH-SY5Y cells with specific antibodies against IRIS-1 and IRIS-2 (purple), and early endosomal antigen 1, EEA1 (specific for endosomes) (green) showed no colocalization between IRIS-1, IRIS-2 and endosomes. Nuclei were stained with DAPI (blue). n=3. Mander's colocalization coefficient between red and green channels (IRIS-1/ 2 - EEA1) is showed on graph (**B**). Scale bars= 10 000 nm. The thickness of each Z-stack is variable between 19.8 to 39.6 µm.

Supplementary figure 4

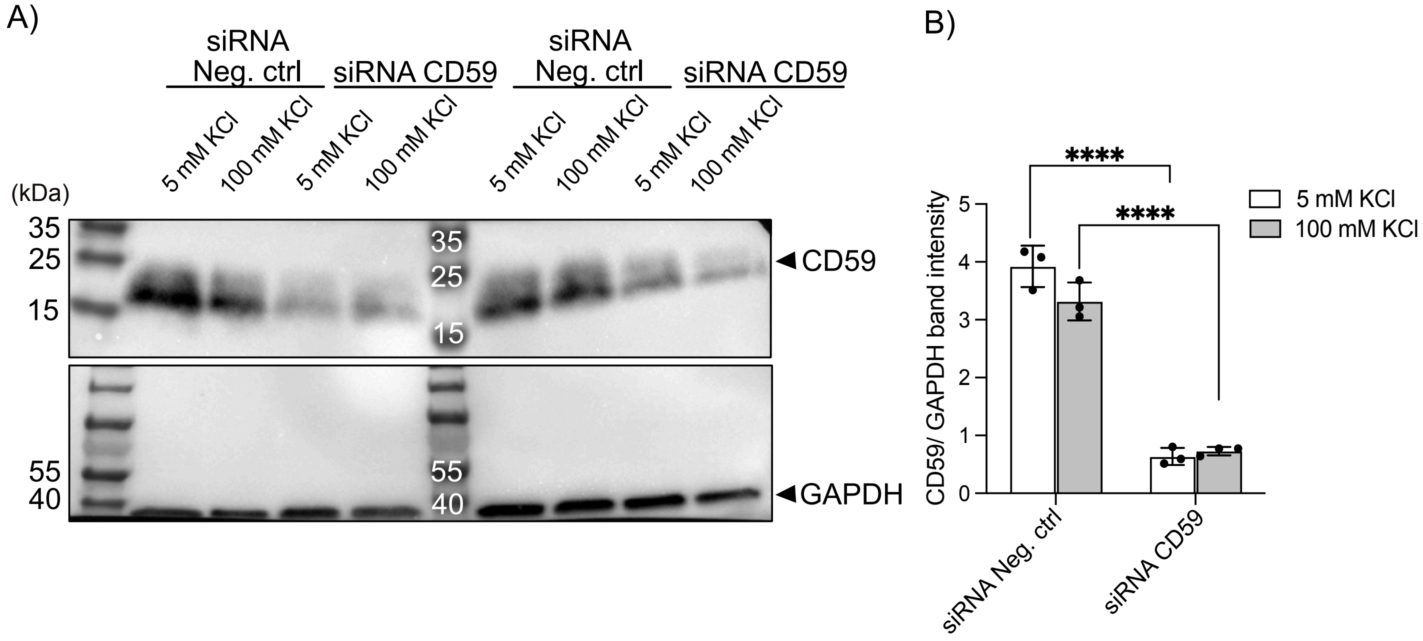

<u>Figure S4:</u> (A) Western blot analysis of protein level expression of CD59 and GAPDH in neuroblastoma cells (SH-SY5Y) treated for 72 hrs with siRNA targeting either CD59 (siRNA CD59), or non-targeting control (siRNA Neg. ctrl) from two independent experiments were run on the same gel. Ratio of bands intensities of CD59 to GAPDH is showed on graph **(B)**. n=3. Statistical analysis was performed using two-way ANOVA. Error bars indicate standard deviations (SD). 5 mM KCL- basal conditions, not stimulating noradrenaline release. 100 mM KCL- evoked conditions.

Supplementary figure 5

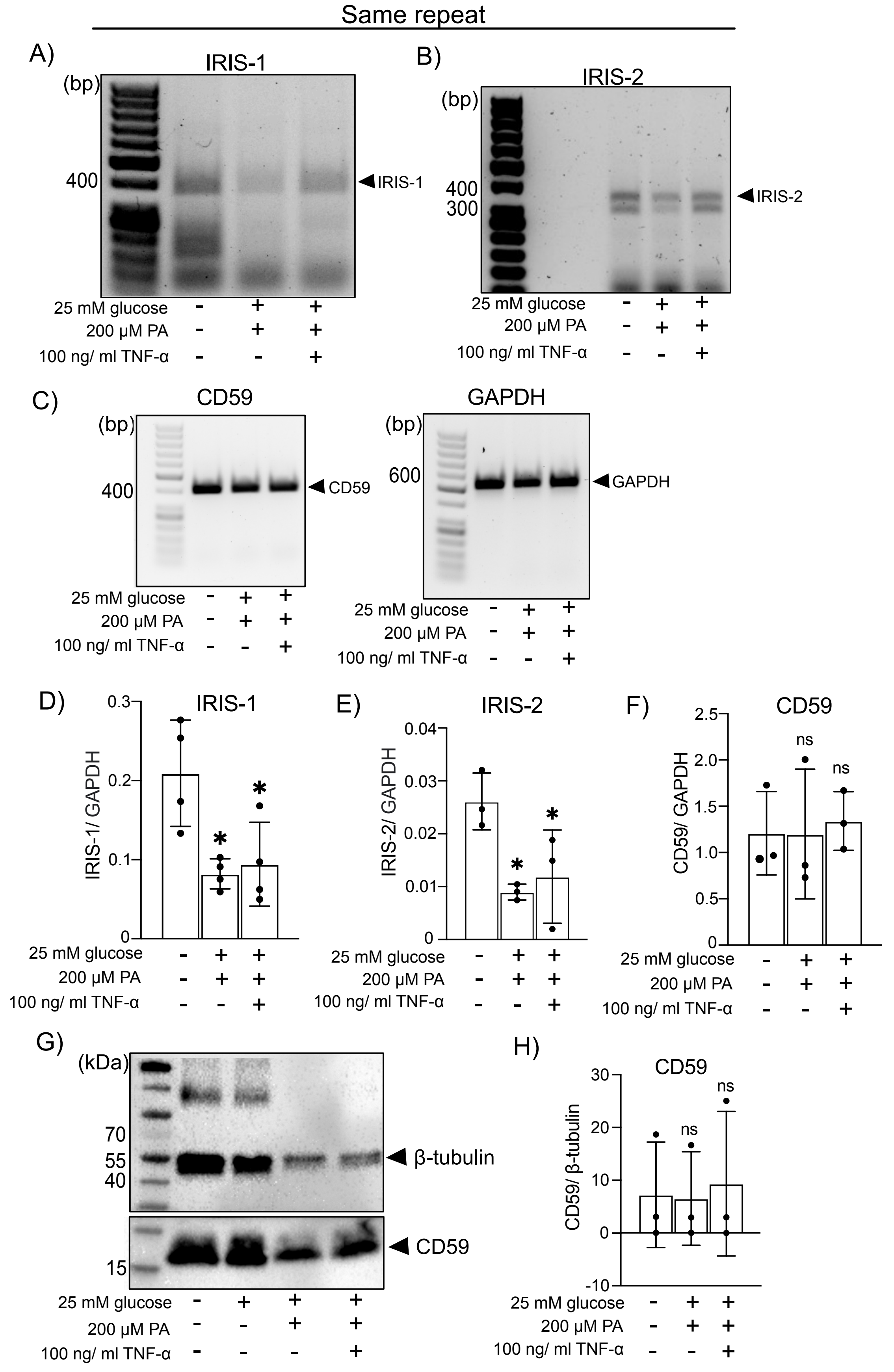

Figure S5: **(A, B, C)** Semi-quantitive RT-PCR analysis of mRNA expression levels of IRIS-1, IRIS-2 and CD59 in SH-SY5Y cells, relative to the reference gene GAPDH upon treatment with high glucose, palmitic acid or cytokine TNF-α, to mimic gluco-/ glucolipo-toxicity and inflammation. Quantification of the gels is showed on **(D)** for IRIS-1, **(E)** for IRIS-2, and **(F)** for CD59. **(G)** Western Blot against canonical CD59, and β-tubulin showed no differences in protein level expression of CD59 upon treatments, quantification in **(H)**. n=4. Statistical analysis for all the panels was performed using 1-way ANOVA, with error bars indicating standard deviations (SD).
